# A Rare Atypical Presentation of Bruns-Garland Syndrome: A Case Report and Review of Pathophysiology and Management

**DOI:** 10.7759/cureus.81692

**Published:** 2025-04-04

**Authors:** Marco A Orsini, Marcos R de Freitas, Marco A Araujo Leite, Wilhelmina N Hauwanga, Aung Ko Oo, Uzma Nezam, Thiago De Mello Tavares, Muskan Garg, Billy McBenedict, Bruno Lima Pessôa

**Affiliations:** 1 Neurology, Federal University of Rio de Janeiro, Rio de Janeiro, BRA; 2 Neurology, Fluminense Federal University, Niterói, BRA; 3 Cardiology, Gaffrée and Guinle University Hospital, Federal University of the State of Rio de Janeiro, Rio de Janeiro, BRA; 4 Faculty of Medicine, Nursing and Health Sciences, Monash University, Melbourne, AUS; 5 Faculty of Medicine, AMA School of Medicine, Metro Manila, PHL; 6 Faculty of Medicine and Stomatology, Tbilisi State Medical University, Tbilisi, GEO; 7 Family Medicine, Universidade Federal de Santa Catarina, Florianópolis, BRA; 8 General Surgery, Lokmanya Tilak Municipal Medical College, Mumbai, IND; 9 Neurosurgery, Fluminense Federal University, Niterói, BRA

**Keywords:** bruns-garland syndrome, diabetic amyotrophy, diabetic lumbosacral radiculoplexus neuropathy (dlrpn), diabetic neuropathy, electroneuromyography (enmg), lumbosacral plexopathy, microvasculitis-induced neuropathy, neurogenic muscle atrophy, peripheral nerve dysfunction, proximal diabetic neuropathy

## Abstract

Bruns-Garland Syndrome (BGS), also known as diabetic lumbosacral radiculoplexus neuropathy (DLRPN) or diabetic amyotrophy, is a rare diabetic complication causing progressive muscle weakness, neuropathic pain, and functional impairment. It primarily affects individuals with long-standing type II diabetes, with an underlying mechanism of microvasculitis-induced ischemic injury to the lumbosacral plexus, leading to axonal loss and neurogenic atrophy. We present the case of a 73-year-old physician with type II diabetes who developed progressive thigh weakness and sensory deficits over seven years. Neurological examination revealed amyotrophy, paresis in the pelvic girdle muscles, and absent deep tendon reflexes. Electroneuromyography (ENMG) demonstrated chronic neuro-radiculopathy with significant axonal loss, and MRI showed bilateral muscle atrophy, edema, and fatty replacement. Unlike typical BGS cases, which present acutely with unilateral symptoms, this patient exhibited a chronic, bilaterally progressive form, highlighting diagnostic challenges. Differential diagnoses included chronic inflammatory demyelinating polyradiculoneuropathy, lumbar spinal stenosis, and neoplastic neuropathies. Management focused on glycemic control, physical therapy, and neuropathic pain management, with consideration of immunomodulatory therapy in severe cases. This case underscores the need for heightened clinical awareness of atypical BGS presentations and the role of electrodiagnostic and imaging studies in distinguishing it from other neuropathies. Early recognition and comprehensive management are crucial to improving outcomes and preventing further functional decline.

## Introduction

Bruns-Garland Syndrome (BGS), also referred to as diabetic amyotrophy, diabetic lumbosacral radiculoplexus neuropathy (DLRPN), or proximal diabetic neuropathy, was first described in the late 19th century. Leyden (1877) and Auche (1890) were among the first to recognize the condition, while Bruns (1890) is credited with the initial detailed description [1,2]. The term "diabetic amyotrophy" was later introduced by Garland in the 1950s, contributing to the current understanding of the syndrome [1]. 

BGS primarily affects individuals with type II diabetes, with the highest incidence occurring during the sixth decade of life [3]. Although it is more common in type II diabetes, owing to longer disease duration and increased comorbidities, it can also be present in type I diabetes [4]. Clinically, BGS is marked by sudden, severe pain, usually in the hip or thigh, often accompanied by muscle weakness of the lumbosacral plexus, and is frequently associated with weight loss, while other constitutional symptoms are generally absent [3]. Risk factors include advanced age, hypertension, peripheral vascular disease, smoking, dyslipidemia, poor glycemic control, prolonged diabetes duration, obesity, excessive alcohol consumption, and the HLA-DR3/4 genotype [5]. Additionally, the condition is more prevalent in males than in females, with a male-to-female ratio of approximately 3:2 [6,7].

The pathogenesis of BGS is thought to involve T-cell-mediated microvasculitis targeting small epineurial and perineurial vessels, leading to ischemic damage of nerve roots and plexuses, which results in nerve dysfunction [3]. Histopathological studies reveal perivascular inflammation with CD4 and CD8 lymphocytes, without vessel wall destruction, along with elevated levels of inflammatory cytokines and membrane attack complexes [3]. The presence of hemosiderin deposition and focal or multifocal axonal loss further supports autoimmune and inflammatory mechanisms driving nerve damage [3].

Histopathological analyses highlight microvasculitic changes and inflammatory infiltrates in affected nerves, emphasizing microvascular ischemia as a key contributor to nerve injury [3]. Imaging studies have provided valuable insights into the extent of nerve involvement, demonstrating widespread damage to the lumbosacral plexus [3]. The aim of this study was to present a rare, atypical case of BGS. By detailing the patient’s clinical features, electrophysiological findings, and imaging results, this study seeks to create awareness of atypical manifestations of BGS. Additionally, through a review of the pathophysiology and management strategies, this study aimed to provide insights into the underlying mechanisms driving BGS and discuss current and emerging therapeutic approaches to optimize patient outcomes.

## Case presentation

A 73-year-old physician with a 40-year history of type II diabetes, managed with insulin for the past 10 years, presented with progressive symptoms over the past seven years. He initially developed weakness and fatigue in the proximal third of his thighs, resulting in significant functional impairment. Information regarding proximal muscle weakness was inferred from the patient's reported difficulty standing up from the ground, rising from low chairs, and walking short distances. The patient also experienced significant muscular fatigue and exhaustion. The patient reported difficulties with ambulation, climbing stairs, walking long distances, and rising from low surfaces. Clinical observation revealed a positive Gowers’ sign when attempting to stand.

On neurological examination, the patient exhibited amyotrophy in the proximal thigh and pelvic girdle muscles, with paresis in the psoas, quadriceps, and gluteal muscles. Deep tendon reflexes were absent in both upper and lower limbs. Sensory testing revealed reduced superficial and deep sensitivity, characterized by hypoesthesia and hypopalesthesia. Electroneuromyography (ENMG) revealed chronic neuro-radiculopathy with significant axonal loss predominantly in the pelvic region (Table 1). F-wave studies showed prolonged minimal latency in the tibial nerves (Table 2). Additional ENMG findings indicated denervation in the gluteal and paravertebral muscles bilaterally (Tables 3, 4). All laboratory findings were normal, including tumor markers and tests for immune diseases and paraneoplastic syndromes. These results were confirmed by both blood analysis and cerebrospinal fluid evaluation. No further tests were performed on the cerebrospinal fluid due to the absence of standard biomarkers (paraneoplastic, immune-mediated disease, tumor).

**Table 1:** Sensory nerve conduction studies

Nerve	Location	Recording Site	Onset Latency (ms)	Peak Latency (ms)	Negative Peak Amplitude (μV)	Positive Peak Amplitude (μV)	Distance (mm)	Velocity (m/s)
Left Superficial Peroneal (Antidromic)	Lateral Leg	Ankle	NR	NR	NR	NR	120	NR
Right Superficial Peroneal Nerve (Antidromic)	Lateral Leg	Ankle	NR	NR	NR	NR	120	NR
Right Sural Nerve (Antidromic)	Calf	Ankle	3.25	4.00	5.80	8.30	140	43
Left Sural Nerve (Antidromic)	Calf	Ankle	3.4	4.20	3.70	4.80	140	41

**Table 2:** F-Wave latency parameters

Nerve	F-Wave Latency (ms)	M-Wave Latency (ms)	F-M Latency Difference (ms)
Left Tibial Nerve - Abductor Hallucis Muscle (AH)	55.9	63.5	7.6
Right Tibial Nerve - Abductor Hallucis Muscle (AH)	64.5	8.3	56.2

**Table 3:** Motor nerve conduction studies

Nerve/Location	Latency (ms)	Amplitude (mV)	Relative Amplitude (%)	Duration (ms)	Distance (mm)	Velocity (m/s)	Area (mVms)	Area (%)
Right Peroneal Nerve - Extensor Digitorum Brevis Muscle (EDB)								
Ankle	4.50	2.30	100	3.90	80		5.0	100
B. Fibular Head	13.22	2.00	83.4	4.00	360	41	4.4	89
A. Fibular Head	15.66	2.10	107	4.00	100	41	4.7	93.4
Left Peroneal Nerve - Extensor Digitorum Brevis Muscle (EDB)								
Ankle	6.52	1.90	100	6.50	80		5.3	100
B. Fibular Head	14.24	1.20	61.8	7.20	340	44	5.6	105
A. Fibular Head	15.62	0.90	78.9	7.00	100	72	1.9	34.9
Right Tibial Nerve - Abductor Hallucis Muscle (AH)								
Ankle	6.08	3.00	100	4.60	80		6.6	100
Knee	17.02	1.90	64	5.20	410	37	5.3	80.7
Left Tibial Nerve - Abductor Hallucis Muscle (AH)								
Ankle	5.08	1.20	100	5.00	80		2.0	100
Knee	16.38	0.80	64	5.80	420	37	1.3	68.3

**Table 4:** H-Reflex latency parameters

Nerve	M-Wave Latency (ms)
Left Tibial Nerve - Soleus Muscle	4.950
Right Tibial Nerve - Soleus Muscle	6.900

**Figure 1:**
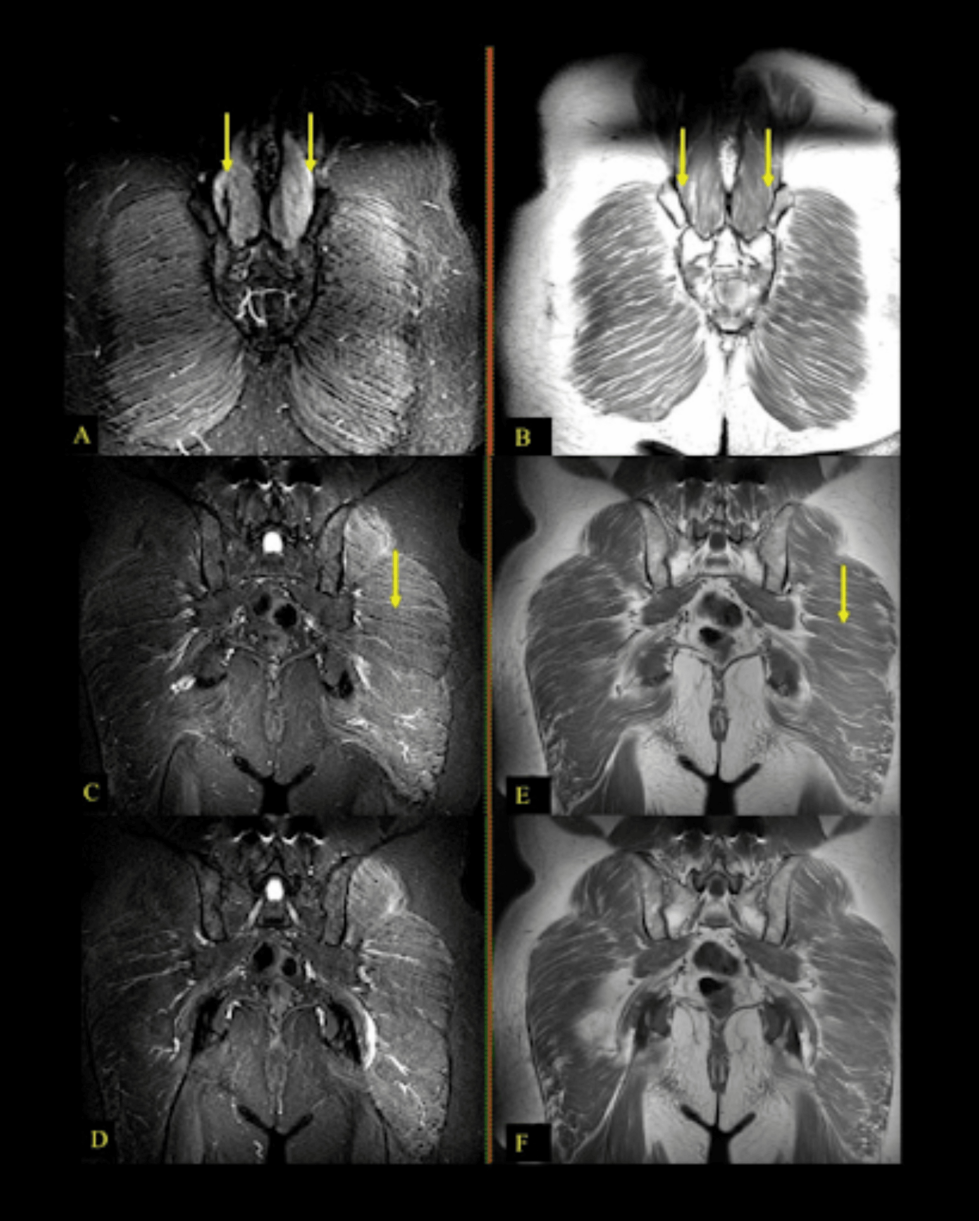
Pelvic MRI findings demonstrating bilateral paravertebral muscle atrophy with edema and partial fatty replacement (A-B: coronal STIR and T1 sequences, arrows). Bilateral gluteal muscle atrophy, more pronounced on the left, with edema and partial fatty replacement (C-F: coronal STIR and T1 sequences).

Pelvic MRI provided further diagnostic clarity. Coronal STIR sequences showed muscle atrophy with edema and partial fatty replacement of the paravertebral musculature bilaterally. Additionally, bilateral gluteal muscle atrophy was observed, more pronounced on the left, along with edema and partial fatty replacement on T1 sequences. Coronal imaging also revealed atrophy of the adductor muscles with partial fatty replacement. Bilateral sciatic nerve involvement, particularly on the left side, was noted, with associated edema (Figure 1).

The patient was treated with Thioctacid 600 mg daily, Metformin 500 mg twice daily, Dapagliflozin 10 mg daily, Gabapentin 300 mg twice daily, and Duloxetine 60 mg daily. Despite diabetic management and motor rehabilitation, the patient showed no improvement in muscle weakness; however, their condition remained clinically stable. The medications provided pain relief but had no effect on muscle strength, which remained unchanged. In most cases of Bruns-Garland syndrome, the prognosis remains guarded. Patients typically experience little to no improvement in muscle weakness, despite clinical management and targeted rehabilitation programs. 

## Discussion

Clinical features

The patient, a 73-year-old male with a long history of type 2 diabetes, exhibited several symptoms that are consistent with BGS. Proximal muscle weakness, particularly in the psoas, quadriceps, and gluteal muscles. Additionally, amyotrophy in the proximal thigh and pelvic girdle muscles is a hallmark feature of BGS. The absence of deep tendon reflexes in both upper and lower limbs further supports the diagnosis, as this is a common finding due to motor nerve involvement. Sensory deficits, including hypoesthesia and hypopalesthesia, though typically mild in BGS, were also present in this case. Electrophysiological findings, such as chronic neuro-radiculopathy with significant axonal loss, prolonged F-wave latencies, and denervation, provide additional confirmation. MRI findings of muscle atrophy, edema, and fatty replacement, particularly in the gluteal and paravertebral regions, further reinforce the diagnosis. However, certain features of this case deviate from the typical BGS presentation. The prolonged disease course, with symptoms evolving gradually over seven years, is unusual, as BGS typically presents with an acute or subacute onset and progresses over months to two years. Moreover, while BGS predominantly affects one side, this patient exhibited bilateral muscle atrophy and nerve involvement, which is atypical. The extent of sensory impairment was also notable; while mild sensory loss can occur in BGS, this patient had more pronounced deficits than expected. 

According to Agarwal et al. [7], BGS presents with severe unilateral pain followed by proximal muscle weakness and atrophy in the thighs and hips, leading to impaired mobility. It may involve a sensory-predominant presentation, but motor involvement is typically more pronounced in neurophysiological evaluations [8]. In rare cases, a painless variant can delay diagnosis [9,10]. The disease progresses through acute, subacute, and chronic stages over months to two years, sometimes advancing to quadriparesis, with severe muscle atrophy causing functional impairment [9]. Systemic symptoms include weight loss, poor glycemic control, and autonomic dysfunction in around 50% of cases, complicating management [11,9].

Jiménez-Ávila et al. [12] reported a 59-year-old diabetic male with sudden lower extremity weakness and loss of sphincter control, where radiographs showed grade II L5-S1 spondylolisthesis, but MRI revealed minimal changes and electrophysiological studies confirmed diabetic amyotrophy. Both that case and the current report involve older diabetic males with poor glycemic control and proximal lower limb weakness and pain; however, while Jiménez-Ávila et al. [12] observed more pronounced sensory deficits, the current case is marked by absent deep tendon reflexes and a positive Gowers’ sign. Moreover, the current case demonstrated characteristic muscular atrophy and fatty replacement on imaging and more detailed ENMG findings, including significant axonal loss, active denervation, and increased F-wave latency, contrasting with the previous identification of lumbosacral plexopathy. 

Diagnostic approach and differential diagnoses

The patients presented with a bilateral involvement, demonstrated by electromyography (EMG) and MRI showing symmetric muscle atrophy, edema, and nerve changes deviating from the typical unilateral pattern of diabetic amyotrophy, suggesting a more diffuse and chronic neurogenic process. A comprehensive diagnostic approach is therefore essential to distinguish BGS from other neuropathies.

Differential diagnoses include DLRPN, chronic inflammatory demyelinating polyradiculoneuropathy (CIDP), amyotrophic lateral sclerosis (ALS), lumbar spinal stenosis, vasculitic neuropathies, Guillain-Barré syndrome, neoplastic lumbosacral plexopathy, infectious radiculopathies, hereditary neuropathies, and nutritional deficiencies [13-15]. For example, while CIDP shares a progressive course and proximal muscle involvement, it typically features distal deficits, preserved reflexes, a relapsing-remitting pattern, and elevated CSF protein [8]. In contrast, ALS presents with upper and lower motor neuron signs, hyperreflexia, fasciculations, and bulbar involvement that are absent in BGS, and lumbar spinal stenosis is marked by neurogenic claudication [16,17].

Electrodiagnostic studies, including EMG and nerve conduction studies (NCS), are crucial to make the diagnosis and rule out differentials. EMG reveals muscle denervation patterns consistent with lumbosacral plexopathy [18], and NCS typically shows reduced amplitudes indicative of axonal neuropathy [13]. In atypical cases, such as painless variants, additional investigations such as nerve biopsy with histopathology, laboratory tests for inflammatory markers, and high-resolution lumbosacral MRI are valuable for excluding structural causes like spinal stenosis or tumors [7,13]. MRI findings may include nerve root and plexus enhancement with T2 hyperintensities, although these are nonspecific. Cerebrospinal fluid analysis usually reveals elevated protein without pleocytosis [19], and while inflammatory markers such as erythrocyte sedimentation rate (ESR) and C-reactive protein (CRP) can be mildly elevated, nerve biopsies (often of the sural nerve) are generally not recommended due to their limited diagnostic yield [20].

Table 5 provides a summary of key electrophysiological parameters used to differentiate BGS from other neuropathies. It includes sections on standard measurements from NCS and EMG, diagnostic patterns from overlapping neuropathies, markers such as sensory nerve action potentials, compound muscle action potentials, and motor unit potentials, as well as findings from F-wave and H-reflex studies. The table also presents insights into emerging diagnostic modalities and biomarkers, offering a comprehensive overview to aid in the accurate diagnosis of BGS.

**Table 5:** Electrophysiological diagnostic parameters and differential features in BGS BGS: Bruns-Garland Syndrome, CMAP: Compound Muscle Action Potential; SNAP: Sensory Nerve Action Potential; LDH: Lumbar Disc Herniation; SS: Sensory Syndrome; TM: Tibial Motor; LN: Lateral Femoral Cutaneous Nerve; MST: Malignant Schwannoma Tumor, NCS: Nerve conduction studies, EMG: Electromyography

Features	BGS	Diabetic Peripheral Neuropathy	Inflammatory Radiculoneuropathy	Structural causes	Infectious causes	Neoplastic causes
NCS	Motor conduction slowing in femoral and obturator nerves and absent or reduced Sensory Nerve Action Potentials (SNAPs) in advanced cases due to axonal damage	Reduced SNAP amplitudes (length dependent) and slowing of motor and sensory conduction velocities	Reduced Compound Muscle Action Potential (CMAP) and SNAP amplitudes (multiple nerve roots) and demyelination causes prolonged distal latency and conduction block	Normal SNAPs mostly and reduced CMAP amplitude in affected motor nerves in Lumbar Disc Herniation (LDH) While SNAPs and CMAPs are normal unless chronic damage in Sensory Syndrome (SS)	Often normal SNAPs, unless coexisting radiculopathy or vasculitis and Severe cases: slowing of motor conduction velocity in Tibial Motor (TM), Lateral Femoral Cutaneous Nerve (LN) shows reduced SNAP amplitudes in affected sensory nerves and Mild slowing of motor conduction due to axonal damage	Reduced CMAP or SNAP amplitudes in compressed or infiltrated nerves and severe axonal loss if nerve destruction occurs in Malignant Schwannoma Tumor (MST), Lyphomas shows Reduced SNAPs or CMAPs in nerves affected by lymphomatous infiltration and combination of axonal loss and demyelination
EMG	Fibrillation potentials, positive sharp waves, polyphasic motor unit potentials (MUPs) in proximal muscles	Distal denervation in length dependent distribution and reduced recruitment in distal muscles	Denervation in widespread distribution, ascending weakness and severe recruitment abnormalities which are multifocal	Denervation changes like positive sharp waves in muscles innervated by affected roots and reduced recruitment in myotomal muscles in LDH SS has similar findings with LDH but may involve multiple myotomes or bilaterally	TM- Denervation changes in muscles innervated by affected roots and polyphasic motor unit potentials (MUPs) due to reinnervation LN-fibrillation potentials and postitive sharp waves in focal areas and polyphasic MUPs and reduced recruitment in chronic cases	MST-Denervation in muscles innervated by compressed nerves and chronic denervation and reduced motor unit recruitment Lymphomas-widespread denervation if multiple nerves infiltrated and chronic denervation with patchy muscle involvement
F-wave studies	Prolonged F-wave latencies in femoral or obturator nerves	Mild prolongation in severe cases of motor neuropathy	Prolonged F-wave latencies with motor root involvement	LDH has prolonged F-wave latency in involved nerve roots and SS has diffuse prolongation of F wave latencies	TM- Prolonged F-wave latencies or absent in advanced cases With same findings in LN	Prolonged or absent F-wave in compressed or infiltrated motor nerves
H-reflex studies	Absent or prolonged H-reflex in quadriceps or adductors	Typically, normal unless existing radiculopathy	Absent or prolonged H-reflex, widespread root involvement	Prolonged or absent H-reflex commonly S1 root in LDH while SS has same findings, bilateral in severe cases	TM-prolonged or absent H-reflex in involved lumbosacral roots and prolonged H-reflex latency asymmetric	Absent or prolonged H-reflex in compressed or infiltrated roots
Somatosensory evoked potentials (SSEP)	Prolonged latencies in sensory pathways (if femoral involvement is severe)	Generally normal	Prolonged latencies due to demyelination or axonal loss	Rarely used in LDH while prolonged latencies or reduced amplitudes in chronic compression in SS	Prolonged latencies in sensory pathways of affected regions	Prolonged latencies due to compressed or infiltrated nerve roots
Pattern of Nerve involvement	Localized to proximal nerves (e.g., femoral, obturator), asymmetric	Symmetric length dependent involvement of distal nerves	Polyradiculopathy, ascending often multifocal	LDH has focal involvement of single nerve root while SS has multi-level root involvement often bilateral	TM-diffuse radiculopathy and asymmetric involvement of cranial/peripheral nerves in LN	Localised nerve compression in MST and diffuse involvement in lymphomas
Citations	[7,19,18,21]	[8,19,5]	[8,19,13,20,22]	[19,20,12,22,23]	[19,10,24,25]	[7,19,20,26,25]

Management strategies for BGS 

For patients with early-stage BGS, managing the condition hinges on optimizing glycemic control and providing supportive care. Medication to manage pain, such as tricyclic antidepressants (TCAs) or serotonin-norepinephrine reuptake inhibitors (SNRIs), is crucial alongside physical therapy and nutritional support to promote recovery. Maintaining improved blood glucose levels aids in the prevention of disease progression, with a particular need to avoid rapid blood glucose reduction in patients with chronic hyperglycemia [11,10]. 

For patients with mild, stable, or non-progressive symptoms, support remains key, as diabetic microvascular complications are the disease drivers, not inflammation. Nutritional support with proteins and micronutrients fosters nerve repair, supported by regular follow-ups. Immunomodulatory treatments such as corticosteroids and intravenous immunoglobulin (IVIG) may be considered in cases with severe, progressive, and unresponsive symptoms, though evidence supporting routine use remains limited [9,19,27]. Other treatments, including aldose reductase inhibitors and neuroprotective agents like alpha-lipoic acid, are still being researched for their efficacy in BGS treatment.

Emerging therapies involving stem cells and gene therapy targeting nerve damage and oxidative stress show promise and may influence treatment guidelines in the future. Effective pain management is essential, with neuropathic medications, including gabapentin and pregabalin, and analgesics providing relief [8]. Non-pharmacologic strategies like physical therapy and cognitive behavioral therapy are also beneficial for managing chronic pain. Pain tends to be more severe in the initial six months but resolves within one to three years in many patients [10]. Physical therapy, focusing on maintaining muscle strength and improving mobility, is particularly important in rehabilitating BGS patients [9,28]. Additionally, lifestyle interventions, including regular physical activity, contribute to better glycemic control and enhanced outcomes for diabetes-related complications [11]. Rehabilitation programs should be tailored to the patient’s symptoms and needs, with a well-balanced diet in support of glycemic control and overall health [8].

Rehabilitation plays a critical role, with functional movement-based exercises benefiting walking metrics, walking speed, and ankle range of motion, while aerobic exercises such as cycling, walking, and swimming improve glycemic control and help alleviate neuropathic symptoms [29,30-32]. Balance- and proprioception-based interventions like yoga and tai chi contribute to improved postural control and proprioception, addressing instability typically seen in diabetic neuropathy [33-35]. Despite variability in patient response and adherence, these rehabilitation therapies show a significant positive impact when consistently applied.

## Conclusions

BGS is a rare diabetic neuropathy that primarily affects the lumbosacral plexus, leading to progressive muscle weakness, sensory loss, and functional impairment. This case highlighted an atypical presentation with a prolonged disease course and bilateral involvement, deviating from the classic acute, unilateral pattern. The patient’s clinical, electrophysiological, and imaging findings emphasized the need for a thorough diagnostic approach to differentiate BGS from other neuropathies, including CIDP, ALS, and lumbar spinal pathology. Early recognition and appropriate management, including glycemic control, symptomatic relief, and rehabilitation, are crucial for optimizing outcomes. While the pathogenesis remains incompletely understood, microvascular ischemia and inflammatory mechanisms are key contributors. Future research on targeted immunomodulatory therapies and neuroprotective strategies may improve treatment efficacy. This case highlighted the importance of multidisciplinary evaluation and individualized management in patients with complex diabetic neuropathies.
